# Addressing the Proximal Causes of Obesity: The Relevance of Alcohol Control Policies

**DOI:** 10.5888/pcd9.110274

**Published:** 2012-05-03

**Authors:** Deborah Cohen, Lila Rabinovich

**Affiliations:** Author Affiliation: Lila Rabinovich, RAND Corporation, Arlington, Virginia.

## Abstract

Many policy measures to control the obesity epidemic assume that people consciously and rationally choose what and how much they eat and therefore focus on providing information and more access to healthier foods. In contrast, many regulations that do not assume people make rational choices have been successfully applied to control alcohol, a substance — like food — of which immoderate consumption leads to serious health problems. Alcohol-use control policies restrict where, when, and by whom alcohol can be purchased and used. Access, salience, and impulsive drinking behaviors are addressed with regulations including alcohol outlet density limits, constraints on retail displays of alcoholic beverages, and restrictions on drink “specials.” We discuss 5 regulations that are effective in reducing drinking and why they may be promising if applied to the obesity epidemic.

## Introduction

Overweight and obesity are global problems, affecting most people in developed countries and a growing proportion of those in the developing world (1). The immediate cause of overweight and obesity is well understood (consumption of calories in excess of energy expended), and the means to reduce overweight and obesity have also been identified (reduce calorie consumption and engage in more physical activity). Yet most people cannot lose weight or sustain the weight loss long-term (2).

Evidence suggests that increased food consumption plays a larger role in the obesity epidemic than does decreased physical activity (3). Many restrictive food-related policy-level interventions to address the obesity epidemic have been proposed but have yet to be adopted broadly, including taxes on low-nutrient foods and beverages, advertising restrictions, and restrictions on fast-food outlets. In contrast, schools are quickly adopting policies to control the nutritional quality of school meals and snacks. Other community-wide policies more readily adopted include increasing access to fruits and vegetables and menu labeling, both of which assume that people will make better choices with more access and relevant information. However, convincing evidence of effectiveness of either of these approaches is lacking (4).

Effective policy interventions to control consumption of alcohol, another substance that, if consumed in excess, can lead to serious health consequences, focus on limiting access to alcoholic beverages by restricting where, when, and by whom they can be purchased and consumed. Although policy lessons from tobacco-use control may also be informative, the parallels between moderate alcohol and food consumption make alcohol a more relevant comparator. Just as moderate consumption of alcohol does not necessarily lead to harm, moderate consumption of low-nutrient foods is also not likely to increase the risk of diet-related chronic diseases; conversely, any use of tobacco is harmful.

The differences between alcohol and food are notable. Alcohol is a controlled substance that is not essential for survival. It is also psychoactive, banned altogether for people under certain ages (21 in the United States), and many of the harms from its consumption are immediate. Although alcohol-related injuries and diseases are related to the total quantity of ethanol consumed in a given period, the relevance for some diet-related chronic diseases is not simply the total number of calories, but also the nutritional value provided in those calories. Despite these differences, alcohol-use control policies offer useful examples of how excess consumption of food might be controlled. Given the magnitude and cost of the growing obesity epidemic, society must go beyond current thinking in addressing the problem.

The consumption of both food and alcohol is related to the social context in which the substance is consumed. Data from multiple countries indicate a close connection between the amount of alcohol consumed by the average drinker and the prevalence of heavy alcohol use in the population (5). For alcohol, the correlation has supported the use of population-level approaches, such as taxation and outlet density control, to tackle problems related to alcohol use. A similarly strong correlation exists between the mean body mass index (BMI) and the percentage of the population that is obese (6,7). Because BMI is a reflection of energy balance, the distribution of BMI across a population indicates that common factors affect the eating behaviors and exercise habits of everyone. Therefore, societal-level measures are likely a relevant and necessary approach to reduce the prevalence of overweight and obesity, much as they are with regard to alcohol use.

We analyzed multiple reviews of alcohol policy (8-16). Given the lack of evidence that the policies would influence rates of obesity, we selected those with evidence of feasibility or effectiveness for alcohol control. Policies had to address issues identified in research as potentially effective for addressing the obesity epidemic. Therefore, the alcohol policies selected for discussion are those that restrict access, discourage impulsive behaviors, limit quantities consumed, or inform consumers about the harms from alcohol misuse. The Table lists various relevant policies, although many of these are unlikely to be politically and socially acceptable for addressing the obesity epidemic. A few of the policies may eventually resonate positively with decision makers and with the public, given their similarity to existing measures used in other fields, namely alcohol- and tobacco-use prevention. We discuss the 5 most promising alcohol-use control policies for translation to obesity control in the current policy climate: 1) density restrictions, 2) rules on display and sale practices, 3) portion control, 4) pricing measures, and 5) warnings about potential harm.

**Table:** Summary of Alcohol-Use Control Policies and Potential Translation to Obesity/Dietary-Related Chronic Disease Control Policies

Existing Alcohol Control Policies	Potential Obesity Control Policies
Limits on alcohol outlet density	Limits on food outlet density
Portion control for servings of alcoholic beverages	Portion control for food servings
Taxes on alcohol	Taxes on foods high in solid oils and added sugars and salt
Prohibitions on drink specials, including all-you-can-drink promotions	Prohibitions on all-you-can-eat food promotions
Alcohol sold only in licensed establishments	Food sold only in licensed establishments, licenses restricted to outlets where food sales comprise >50% business
Alcohol sold in gas stations cannot be displayed near cash register	Prohibition of displaying high-sugar/high-fat foods as impulse buys, near cash registers, and on ends of aisles
Counter-advertising	Campaigns against low-nutrient foods
Warning labels on alcohol	Warning labels on processed food high in solid oils and added sugar and salt
Prohibitions of drinking on the job	Limits on food availability at the workplace; other incentives/services for weight control; regulations on food accessibility
Quality control of alcohol (percentage alcohol per drink)	Quality control or naming of food by percentage of fat and sugar content
Limiting hours of service	Reducing hours of outlets predominantly promoting items with low nutrient value
Prohibiting drive-through alcohol sales	Drive-through service limited
Prohibitions on driving and drinking	Prohibitions on driving and eating
Server training requirements	Food servers trained in portion control and promoting healthier alternatives
Prohibitions on sales to youth younger than age 21 y	Prohibition of sales of foods restricted in schools (selected items high in fats and sugars) to youth younger than age 18 y

## Density Restrictions

### Alcohol policy

Density regulations limit the number of licenses that are issued to permit the sale of alcohol. Places with a high alcohol outlet density have higher rates of violence, injuries, and drunk-driving fatalities than those with a low density of such establishments (8,13). Furthermore, where the number of alcohol outlets increases, so does the level of drinking (17,18). Similarly, where alcohol outlet density has been reduced, the health consequences associated with problem drinking have decreased (19,20).

Density restrictions work in 2 ways. First, they reduce the frequency of cues related to drinking. Second, density restrictions make alcohol less accessible, effectively increasing the cost of getting it. When the costs of drinking go up, drinkers (including alcoholics) will moderate consumption (21).

### Relevance to obesity control

Easy access to foods high in calories and low in nutritional value is a stimulus of hunger and the desire to eat (22). However, proximal cues are often perceived in ways that are difficult or impossible for people to recognize (23). People often experience the illusion that their desires for food develop solely from within, based on true need, rather than being stimulated by external cues (24).

The ubiquitous presence of food undermines people’s ability to control impulsive eating behaviors, which are triggered by a physiologic reflexive dopamine reaction (25). Limiting the density of food outlets, in particular outlets that primarily sell food items high in calories and low in nutritional value, could help reduce consumption of such foods. Density limits could be applied to food outlets by type of food sold; a few localities already have ordinances in place that restrict the opening of new fast-food outlets (26). Such ordinances could be expanded to cover convenience stores or specialty food outlets devoted to the sale of foods high in discretionary calories (eg, doughnut shops, ice cream parlors).

Licensing and outlet density restrictions may also help curb sales of food in places that are not primarily food outlets. Licenses are typically required only for food outlets that that sell perishable food. Outlets selling food that doesn’t spoil, such as highly processed candies, salty snacks, sugar-sweetened beverages, and foods that do not need refrigeration, are generally not required to be licensed or inspected. Establishments with vending machines typically do not obtain food licenses. Consequently, hardware stores, bookstores, worksites, gas stations, schools, and other nonfood outlets and public venues are increasingly likely to sell nonperishable foods or have vending machines (27).

Although restricting exposure to low-nutrient snack foods may help control obesity by reducing the appetitive stimulation they generate (28), a question arises as to whether limiting the accessibility of these types of foods places an undue burden or cost on people who are not overweight and not easily tempted (ie, “moderate” eaters). The ubiquity of unhealthy food was lower before 1980, and no evidence seems to suggest that consumers were unduly burdened. Today, a minority of people in developed countries could be considered “moderate” eaters, if normal weight for height is a marker of moderate eating (1).

Much attention is devoted to increasing the availability of fresh fruits and vegetables in low-income areas designated as “food deserts,” defined as areas whose residents live more than one-half mile from a supermarket and do not have access to a vehicle (29). Less than 5% of the American population lives in areas than can be classified as food deserts, yet 67% are overweight or obese. Nevertheless, just as in some localities all establishments that serve alcohol are required to serve food and nonalcoholic beverages, a policy that requires food outlets to have some minimum number of healthy options may be a policy alternative. However, obesity is less the result of eating insufficient healthy foods and more the consequence of eating too many unhealthy ones. Restrictions on unhealthy foods are more promising than promotion of healthy foods in controlling obesity (30,31).

## Display and Sales Restrictions

### Alcohol policy

Many efforts in the United States have attempted to reduce the impulsive consumption of alcohol and drinking while driving. In California gas stations, the sale of beer is prohibited from iced barrels or from temporary displays placed within 5 feet of the front door or the cash register (32). Although the evidence on the effectiveness of measures regulating displays and presentation is patchy, research shows that after New Mexico banned drive-through alcohol outlets in 1998, sales of alcohol decreased and rates of alcohol-use–related fatalities continued to decrease (33,34). In most localities, to discourage its immediate consumption, alcohol cannot be sold through a drive-through window.

### Relevance to obesity control

The restrictions used for alcohol control could be applied to food outlets to discourage impulse purchases of low-nutrient foods. Vendors pay supermarkets slotting fees to put their products where they are easily noticed, such as at eye level, on end aisles, and on special floor displays, which block aisles and require customers to stop and take notice. Saliently placed products sell in greater volume than in less salient areas; end-aisle displays account for 30% of all supermarket sales (35). To counter the influence of salience, foods that are high in calories and low in nutritional value may be restricted to locations such as the back of store, behind the counter, or at locations other than end aisles or eye-level displays. Regulations could also be established concerning which foods may be displayed at the cash register or in other prime locations of a grocery store or other food outlet, as well as which foods can be sold through a drive-through window.

## Portion Control

### Alcohol policy

Alcoholic beverages are classified by their percentage of alcohol content, and the US government defines a standard drink as containing 0.6 oz of alcohol. Therefore, based on their concentration of alcohol, standard drink sizes are 12 ounces for a glass of beer, 5 ounces for a glass of wine, and 1.5 ounces for a “shot” of 80-proof liquor. These standard portion sizes have been established to allow people to estimate their risk of inebriation based on the number of drinks consumed. In 8 American states, laws prohibit selling larger quantities per drink without also increasing the price (36). Furthermore, incentives such as lowered taxes and relaxed regulation of sales have been extended to alcoholic drinks with lower alcohol content such as “near beer,” an approach that has been effective in reducing alcohol-related harms (8).

### Relevance to obesity control

Larger servings of food have been associated with higher energy intake, regardless of serving method and the characteristics of individual eaters (37-39). The increase in portion sizes during the past 3 decades in the United States is well documented (40) and problematic because people tend to underestimate portion size (41) and cannot easily judge how much they have consumed (42).

Although menu labeling that specifies the caloric content of specific dishes is required in US restaurants with 20 or more outlets, choosing healthy foods in the appropriate quantity is still difficult for people (43). Default serving sizes by volume or weight for all foods could be established internationally. The US Food and Drug Administration has created Reference Amounts Customarily Consumed (RACCs) based on what people ate in the 1980s (44). Although RACCs do not provide precise calorie counts, using these as guidelines would help reduce overall consumption. No specialized training or equipment other than a measuring cup or a kitchen scale would be required for adherence to portion control standards.

Default portion sizes could be applied to all foods, but maximum serving sizes should be set for foods that are high in calories and low in nutritional value, such as sugar-sweetened beverages and deep-fried foods. Portion control is intended to help people gauge how much they have eaten. Some people who are large or very active may find a standard portion insufficient. Just as people can order more than 1 drink, people would be free to order more than 1 portion.

## Pricing Measures

### Alcohol policy

Problem drinkers (including binge drinkers and heavy chronic drinkers) tend to choose cheaper alcoholic beverages because they seek to maximize ethanol intake for the money they spend (11,45). In the United States, the top 10% of drinkers spend approximately $0.78 per drink compared with $4.75 per drink for the bottom 50% of drinkers (46). This fact provides a strong rationale for the use of measures that increase the price of the cheapest drinks. Policies used abroad may offer useful lessons in this area. For example, in Poland, Luxembourg, and Belgium, establishments that sell alcoholic beverages for off-premise consumption are subject to a general ban on below-cost sales, which also applies to sales of alcohol (47). Many Canadian provinces use “social reference pricing,” which sets minimum prices per unit of pure ethanol to ensure that alcohol prices do not fall below a certain threshold, making alcoholic beverages more expensive and reducing their consumption among problem drinkers. In Germany, the “Apple Juice” law states that in outlets that sell alcohol for on-premise consumption, at least 1 alcohol-free beverage must be cheaper than the cheapest alcoholic beverage available. In Switzerland in some cantons (ie, provinces), all restaurants are obliged by law to offer at least 3 nonalcoholic drinks that are cheaper than the cheapest alcoholic beverage of the same quantity (47).

Restrictions on on-premise alcohol price promotions are also common. Many US states and localities prohibit “specials,” such as “all-you-can-drink” nights and “ladies drink free” nights (36). These prohibitions discourage drunkenness, binge drinking, and other alcohol-related problems (48).

### Relevance to obesity policy

Foods that are high in calories and low in nutritional value could be subject to a higher tax. Fruits and vegetables could be required to be less expensive than foods such as candies, cakes, or French fries that can exacerbate or increase the risk of chronic diseases. Restrictions on “all you can eat” one-price buffets should also be considered, because the more people eat, the lower the cost per calorie. This is an incentive to overconsume. Buffet costs could be based on the weight of the food purchased, and buffet items could be served in controlled portion sizes to reduce the risk of people overeating. In supermarkets, specials such as “10 for $10” or “2 for the price of 1” can be prohibited for foods with a low-nutrient profile.

## Warning Labels and Counter-Advertising

### Alcohol policy

Warning labels on alcohol bottles and tobacco packages have been moderately effective in increasing awareness of the respective risks of alcohol and tobacco use, although the effect of labeling on actual drinking and smoking behavior remains contested (49,50). Nevertheless, the value of warning labels as a means to improve awareness and understanding and to shift perceptions and attitudes on the risks of particular goods is undeniable. Counter-advertising as a means to reduce consumption has proven effective in modern times for tobacco control (51). In the mid-19th century, the temperance movement had a campaign maligning alcohol as “demon rum” and widely circulated materials about the harms, both spiritual and physical, associated with alcohol use. Historians have credited these efforts with contributing to a precipitous reduction in alcohol consumption (52).

### Relevance to obesity control

Although nutrition labeling is mandatory in the United States, warnings are not. The use of traffic light labeling — placing red, yellow, and green circles to respectively signify large, medium, and small amounts of fats, sugars, and salt — increases the frequency with which people can identify healthier products (53). Whether this information ultimately changes consumer behavior sufficiently to reduce overweight and obesity is still unknown. Signs and symbols to convey that consuming certain foods in excess could increase the risk of heart disease (eg, high-fat foods) or other chronic diseases (eg, hypertension, diabetes) may help consumers make choices at the point of purchase. Because warning symbols have never been used to help people avoid a specific chronic disease from food consumption, research in this area is warranted.

A limited number of mass media campaigns exist that discourage people from eating too much or that highlight the importance of refraining from eating foods with little nutritive value. We are not aware of formal evaluations of these campaigns, but they may have an effect if they are salient and reach a large number of consumers.

## Conclusion

Alcohol-use control policies have not eliminated problems related to alcohol use but have kept the problems under control in localities where the policies are strictly implemented and enforced. However, alcohol policies, especially those seen to infringe on individual choice (such as restrictions in outlet density) or to negatively affect moderate drinkers who do not cause harms (such as excise taxation) have been controversial. Over time, many of these measures have become widely accepted and do work in curbing problems related to alcohol use.

Compared with mortality attributed to alcohol consumption, death rates attributable to overconsumption of food and poor diet are considerably higher (54). However, systematic efforts to address overweight and obesity are still in their infancy. One reason the obesity epidemic has not been stemmed is because of the direct effect that policies regulating the food environment have on industry; reducing rates of obesity requires that people eat less of certain kinds of foods, leading to businesses selling fewer of these products and, thus, to potentially lower profits. Consequently, resistance to the types of restrictions proposed here will likely be high. In particular, policies such as density limits that could lead to business closures might be the most difficult to pass, but they could eventually be achieved by banning new outlets and not reissuing licenses once an outlet closes. However, some of the other policies mentioned, such as standardizing portion sizes or introducing measures that raise the price of high-calorie, low-nutrient foods, could be adopted quickly and could have a positive effect on profits if outlets sell smaller quantities at the same or higher price.

The acceptability of restrictive policies for people may be low if people perceive that they are paying higher prices for less food. However, this may not be the perception if reductions in the quantities of energy-dense, low-nutrient foods are matched by increases in the volume of nutrient-rich low-energy foods. Altering portion sizes should have the greatest benefit for people with lower ability to compensate.

As the prevalence of obesity and its associated health-related costs have increased, the need for society to take stronger action is becoming apparent. Just as regulating alcohol accessibility has been effective in reducing problem drinking, regulating food accessibility is promising for controlling the obesity epidemic. Policies to address obesity need to be multipronged, incorporating a mix of approaches that include restrictions in access to problem foods, reducing impulse purchases, using point of purchase warnings, and attempting to control portion sizes.

In the early 19th century, the temperance movement, working alongside the development of social abstinence clubs, used a multitude of strategies to reduce drinking. They effectively reduced the density of alcohol outlets, initially by subsidizing alcohol-free taverns where owners said they would not make profits unless they sold alcohol. They encouraged the banning of alcohol from workplace environments and from retail shops. They disseminated extensive negative communications about alcohol’s harms. In 1 decade, from 1830 to 1840, the consumption of alcohol dropped more than 50%, from nearly 4 gallons to less than 2 gallons per capita (52). Although different social mores were at play than exist today, radical changes made a rapid dent in alcohol use. In the face of the emerging challenge of overweight and obesity, alcohol control policies could be important models to follow.
